# Is Cross-Reactive Immunity Triggering COVID-19 Immunopathogenesis?

**DOI:** 10.3389/fimmu.2020.567710

**Published:** 2020-10-15

**Authors:** Alberto Beretta, Martin Cranage, Donato Zipeto

**Affiliations:** ^1^Solongevity Research, Milan, Italy; ^2^Institute for Infection and Immunity, St George's, University of London, London, United Kingdom; ^3^Laboratory of Molecular Biology and Virology, Department of Neuroscience, Biomedicine and Movement Sciences, University of Verona, Verona, Italy

**Keywords:** COVID-19, SARS-CoV-2, antibody-dependent enhancement, immunopathogenesis, cross-reactivity, human coronaviruses

## Abstract

The serological responses to both SARS-CoV-1 and SARS-CoV-2 virus have some unique characteristics that suggest cross-reactive priming by other human coronaviruses (hCoVs). The early kinetics and magnitude of these responses are, in some cases, associated with worse clinical outcomes in SARS and COVID-19. Cross-reactive hCoV antibody responses have been detected in both SARS and COVID-19 patients. There is also evidence that pre-existing T cell immunity to common cold coronaviruses can prime the response to SARS-CoV-2. Studies in non-human primates show that SARS-CoV-1 S-protein vaccine-induced antibodies are associated with acute lung injury in macaques challenged with SARS-CoV-1. Here we discuss the potential of cross-reactive immunity to drive the immunopathogenesis of COVID-19 and its implications for current efforts to develop immune-based therapies and vaccines.

## Introduction

The new SARS-CoV-2 pandemic has overwhelmed the world with its high contagiousness and range of severity, presenting from asymptomatic infection or mild symptoms to severe acute respiratory syndrome with severe morbidity and mortality. The lack of pre-existing immunity is thought to be one reason for the rampant spread of the virus.

The duration and nature of immune responses to SARS-CoV-2 infection are not yet fully understood. Many public health responses to COVID-19 outbreaks are based on the assumption that the infection will result in a protective immune response of undefined duration. However, correlates of immunity to SARS-CoV-2 may be complicated by the existence of a pre-existing immunological memory to other human coronaviruses. In addition to SARS-CoV-2, six human coronaviruses (hCoVs) are known: four seasonal coronaviruses (hCoV-229E, -NL63, -HKU1, and -OC43) which cause mild upper respiratory diseases, and the two most recently discovered viruses, SARS-CoV-1 and MERS-CoV, originating from recent zoonotic events. It is generally assumed that humans are immunologically naive against SARS-CoV-2 and would show a primary immune response to the infection; however, this could be an oversimplification. The possibility that endemic coronaviruses may influence the dynamics of SARS-CoV-2 infection in a way seen among other endemic infections and emerging new variants or viruses should be considered (1, 2).

On the one hand, pre-existing cross-reactive responses to particular epitopes may prime beneficial protective responses, and on the other hand, they may prime immunopathology through mechanisms such as antibody-dependent enhancement (ADE) of infection, whereby immune complexed virus may be taken up into cells that lack the ACE2 receptor as shown for other hCoVs at least *in vitro* (3, 4).

In the cases of SARS-CoV-1 and MERS, experimental vaccine-induced antibodies have been associated with an exacerbated induction of inflammatory cytokines, which is one of the main clinical features in severe COVID-19 patients (5, 6). The picture is further complicated by the intrinsic features of SARS-CoV-2. Blanco Melo et al. (7) compared the host transcriptional response to SARS-CoV-2 with other respiratory virus infections to identify signatures that may be underlying the biology of COVID-19. They demonstrated induction of an aberrant overall transcriptional response to SARS-CoV-2 where, despite virus replication, the host fails to launch a robust IFN-I and -III response while concurrently inducing high levels of chemokines required to recruit effector cells. It is therefore of paramount importance to understand to what extent, if any, the adaptive immune response to SARS-CoV-2 can contribute to virus-induced immunopathogenesis. In this paper we consider the available evidence on the role of cross-reactive immunity in the induction of COVID-19 immunopathogenesis, and we discuss the implications for current efforts to develop immune-based therapies and vaccines.

## Unconventional Antibody Responses Associated With Disease Severity in COVID-19, SARS and MERS Patients

Canonical antibody responses to novel microbial antigens are characterized by the early appearance of IgM followed by IgG and IgA antibodies with an isotype switch pattern typical of a primary infection. In the case of SARS-CoV-2 infection, IgG specific for the receptor-binding domain (RBD) of the S1 subunit of the Spike protein can effectively neutralize viral entry into cells expressing ACE2 resulting in the control of viral replication *in vitro* and in animal models of disease. Rapid and potent virus neutralizing antibody response should therefore be associated with viral clearance and disease control.

In contrast to this expectation, several studies on the early serological response to SARS-CoV-2 have revealed unconventional patterns of seroconversion similar to those of secondary immune responses and an unexpected association between early and potent antibody responses and disease severity. This seroconversion pattern is suggestive of the presence of some degree of cross-reactive immunity that could be induced by previous encounters with common human coronaviruses.

An unconventional pattern of seroconversion to SARS-CoV-2 infection was revealed by the first large serological investigation of COVID-19 patients reported by Long et al. (8) which showed three types of seroconversions: IgM before IgG, synchronous IgM and IgG, and IgM after IgG seroconversion. Most patients showed the last two patterns. Also noteworthy was the finding that patients with severe condition had higher IgG and IgM titers than patients with non-serious conditions.

Similar results were reported by Tan and coauthors (9) who showed that while IgM antibodies appeared simultaneously to IgG in severe and non-severe cases, IgG appeared earlier in severe cases. An earlier IgG seroconversion pattern was also reported by Pan et al. (10). In a study that assessed the antibody response to the RBD of the SARS-CoV-2 S1 subunit, Suthar et al. (11) found that a majority of COVID-19 patients developed class-switched IgG responses specific for RBD, and also IgM and IgA titers lower than IgG indicating that antibody class-switching to IgG occurs early during acute infection. Tan et al. (9) reported that IgM and IgG responses appear earlier and with higher titers in severe patients than in non-severe patients while a significantly higher viral clearance rate was more frequent in weak than in strong responders. A stronger antibody response was associated with delayed viral clearance and increased disease severity in three additional independent studies (9, 12, 13).

Similar observations were also reported following SARS and MERS infections. Zhang et al. (14) described in patients with SARS-CoV-1 that the production of virus-neutralizing antibodies (nAb) in the fatal cases was surprisingly faster than in recovered cases and reached the highest levels on the 15^th^ day after the onset of symptoms, while nAb levels in recovered patients were still increasing. Peak nAb activities were reached within 20 days in recovered patients compared to 14.7 days in patients who subsequently died.

Another study by Ho et al. (15) reported that SARS-CoV-1 early serological responders (antibody detectable within 2 weeks) had a higher mortality rate (29.6 *vs.* 7.8%) and were more likely to be over 60 years old. The authors also reported that the disease was more likely to be of short duration in RT-PCR positive but seronegative patients. In SARS-CoV-1 patients, at week 2, consistent clinical progression, shifting radiological infiltrates, and reversed viral-load profile were associated with disease worsening, which did not correlate with uncontrolled viral replication but rather with immunopathological damage (16). In MERS cases, Ko et al. (17) reported that disease severity was correlated with both seroconversion rate and peak antibody levels, while Okba et al. (18) reported a strong response to severe infections compared to limited or absence of seroconversion in asymptomatic and mild cases.

There is also evidence of an early IgA response associated with disease severity. Using an RBD specific IgA, IgG and IgM test, Huan Ma et al. (19) reported that 4–10 days after symptom onset, the IgA test exhibited the highest positive diagnostic rate. Yu et al. (20) reported IgA seroconversion at day 2 after the symptoms onset, while IgM and IgG appeared at day 5 and the relative levels of IgA and IgG were significantly higher in severe patients compared to non-severe patients.

Cervia et al. (12) reported that patients with severe COVID-19 had a highly significant increase in SARS-CoV-2-specific IgA and IgG serum titers depending on the duration of symptom onset, and regardless of patient age and comorbidities. Very high levels of SARS-CoV-2-specific serum IgA were associated with severe acute respiratory distress syndrome (ARDS).

The apparently early serum IgA response to SARS-CoV-2 is particularly puzzling as in primary encounter with a new antigen an IgM response would be expected to appear first. These preliminary reports should be interpreted cautiously however, as IgM assays can be insensitive and are frequently configured to avoid aberrant cross-reactivity due to the polymeric nature of IgM. It is also possible that total serum IgA levels may be raised in COVID-19, a possibility that requires further investigation. It would be informative to study serum for the presence of multimeric IgA that is normally detectable at only low levels but which could in principal be elevated in COVID-19 due to mucosal damage.

The caveats above notwithstanding, taken together, these early reports provide evidence of an early antibody response associated with disease severity and the possibility of a priming effect due to a previous non-SARS-CoV-2 infection.

## Human Coronavirus Cross-Reactive Antibody Responses in SARS and COVID-19 Patients

In SARS-CoV-1 the reasons why some patients mount an antibody response faster than others are still unknown. The correlation between advanced age and early response with higher titers is indicative of a priming effect from existing endemic strains (15). Similarly, higher plasma nAb titers and spike-binding antibodies have also been observed in elderly and middle-age patients affected by COVID-19 (21). An increase in preexisting IgG antibody titers for human coronaviruses OC43, 229E, and NL63 was observed in SARS patients. In addition, SARS patients without prior exposure to SARS CoV-1, who had OC43 and 229E infections, showed an increase in antibody titers specific to the previously encountered hCoV but not to SARS CoV-1 (22). Acute/convalescent paired samples of SARS patients displayed a more than 4-fold increase in antibodies to hCoV-229E, -NL63, and/or -OC43 (23).

Guo et al. (24) tested the reactivities of human plasma positive for antibodies against NL63, 229E, OC43, HKU1, and SARS-CoV-1, on a recombinant SARS-CoV-2 nucleoprotein (rNP) and found no cross-reactivity. A strong cross-reactivity against SARS-CoV-2 rNP was observed using SARS-CoV-1 positive human plasma (25). However, the consequences of anti-SARS-CoV-1 antibody cross-reactivity in SARS-CoV-2 patients are probably negligible given the very low prevalence of SARS-CoV-1 infection.

Evidence of SARS-CoV-2 and HCoV cross-reactive antibody responses were reported by Nguyen-Contant et al. (26) who studied two cohorts of SARS-CoV-2 unexposed subjects and a cohort of COVID-19 convalescent patients. In unexposed subjects approximately 10% had IgG that bound SARS-CoV-2 S2 but not S1 or the RBD, and 4% had IgG against the SARS-CoV-2 nucleocapsid (N) protein, which is highly conserved among coronaviruses. The levels of IgG against S, RBD, S2, and N were markedly higher in convalescent subjects than unexposed subjects, indicating strong induction of these Abs by SARS CoV-2 infection. In convalescent patients, titers of IgG were higher against the S protein of the HCoV OC43 but not 229E, suggesting a more relevant serological cross-reactivity between betacoronaviruses. Unexposed subjects exhibited memory B cell (MBC) reactive to the S proteins of OC43 and 229E. MBCs reactive to SARS-CoV-2 S, RBD, and N were not found, indicating a very low frequency of these cells in unexposed subjects. In contrast. the vast majority of COVID-19 convalescent subjects had circulating IgG MBCs reactive to the SARS-CoV-2 S1, RBD, and S2 suggestive of a strong induction by SARS-CoV-2 infection of MBCs reactive to both novel and conserved regions of the S protein. The authors suggested that concurrent early production of virus-specific IgM and IgG in response to SARS-CoV-2 infection might be mediated by the stimulation of both IgG MBCs as well as naïve B cells (8, 27–29).

Further substantial evidence of the role played by a cross-reactive antibody response against hCoVs came recently from a study by Yonger et al. (30) in a cohort of SARS-CoV-2 infected children who had multisystem inflammatory syndrome (MIS-C) and showed broadly elevated IgG responses to other coronaviruses.

More recently, the results of an exhaustive profiling of SARS-CoV-2 humoral responses in a cohort of 22 patients reported by Atyeo et al. (31) indicated a skewing of the antibody response to the N protein in patients who subsequently died and a reciprocal skewing toward S in patients who survived. Similar findings were reported by Sun et al. (29). Since both S and N show some degree of homology among coronaviruses, with N being the most conserved, identifying the potential cross-reactivity of S and N antibody responses can help clarify their immunopathogenic role.

An additional feature of the antibody response to SARS-CoV-2 is the rapid recruitment of B cells expressing a limited subset of V genes, together with extensive activation of IgG and IgA subclasses without significant somatic mutations (32).

Therefore, early antibody responses to SARS-CoV-2 can be sustained by both memory B cells primed by previous hCoVs and naïve B cells rapidly recruited from exposure to SARS-CoV-2, and the net result of the balance between the two responses may influence the clinical outcome of the infection.

Gorse et al. (33) observed that functional neutralizing antibodies are not stimulated as frequently as binding antibodies. Lv et al. (34) showed that although cross-reactivity in antibody binding to the spike protein is frequently observed, viral cross-neutralization is uncommon, which is suggestive of a non-neutralizing antibody response to conserved S epitopes with the potential to induce antibody-dependent enhancement.

## Antibody-Dependent Enhancement and Immunopathogenesis

SARS-CoV-1 antibody-dependent enhancement (ADE) of infection was first identified by Yang et al. (35) and was hypothesized to be the reason for such a high mortality rate in China. The priming strains were considered to be human coronaviruses known to cause mild infections such as 229E. ADE is a mechanism by which Dengue viruses exploit humoral antiviral immune responses to infect the host’s cells. ADE has also been well documented in cats infected with the Feline Infectious Peritonitis Coronavirus (36–38), in which disease severity is increased following previous immunization against this virus.

Wang et al. (4) found that antisera against SARS-CoV-1 neutralized SARS-CoV-1 infection at high concentrations, whereas highly diluted antisera significantly increased infection and induced higher apoptosis levels. SARS-CoV-1 ADE was found to be primarily mediated by diluted spike-specific antibodies rather than nucleocapsid specific antibodies.

In the same study, sera from patients that subsequently died induced a strong increase in IL-8 and MCP-1 by *in vitro* polarized monocyte-derived macrophages. In contrast, sera from recovered patients had no effect on cytokine production, suggesting that the two patient groups may present different antibody populations.

In experiments conducted using primary human macrophages, Yip et al. (39) evaluated the effects of anti-Spike antibodies on susceptibility to SARS-CoV-1 infection. Anti-Spike antibodies increased the infection of monocyte-derived macrophages by replication-competent SARS-CoV-1, as well as Spike-pseudotyped lentiviral particles (SARS-CoVpp). However, ADE-induced macrophage infection did not support productive viral replication. In addition, purified anti-Spike IgGs were sufficient to enhance infection, but not other soluble factors present in the mouse immune sera, providing strong evidence that IgG-mediated ADE can cause infection of human macrophages by SARS-CoV-1. Similar results were reported by Iwasaki and Yang (40) who showed that the internalization of virus–antibody immune complexes promotes inflammatory processes and tissue lesions by activating myeloid cells *via* FcRs. The virus introduced into endosomes through this mode will activate the RNA-sensing Toll-like receptors (TLRs) TLR3, TLR7, and TLR8.

Of particular concern for ADE induction is a mutation in the RBD of SARS-CoV-2 S1 subunit (D614G) which was recently reported by Korber et al. (41). The mutation, which is now the prevalent pandemic form in many countries, may have originated either in China or Europe and has spread very rapidly in Europe and the rest of the world. The authors suggested two possible effects of this mutation on the viral phenotype: (a) a reduction of S1–S2 subunit interaction, resulting in increased shedding of S1 from viral-membrane-bound S2 and (b) a potential induction of ADE: the D614G mutation is located deep within an immunodominant linear epitope of the SARS-CoV-1 Spike (S597–603). This peptide has been recognized as a major immunodominant epitope of SARS-CoV-1 by analyzing sera from convalescent patients infected during the 2001 outbreak. The S597-603 peptide reached 64% serological reactivity and induced long term B-cell memory responses. In rhesus macaques, S597-603 specific antibodies mediate both *in vitro* and *in vivo* antibody-dependent enhancement of SARS-CoV-1 infection through an epitope-sequence dependent mechanism (4). More importantly, the ADE target peptide embraces the SARS-CoV-2 D614 site and is identical to the corresponding region in SARS-CoV-1. As noted by Wang et al. this epitope is located near the RBD, and antibody binding can mediate a conformational change in Spike that enhances the interaction between RBD and ACE2 inducing the enhancement effect.

Antibodies may induce immunopathology also through mechanisms other than ADE. For example, it has long been known that IgG antibodies to respiratory syncytial virus (RSV), detectable in nasopharyngeal aspirates of infants hospitalized with RSV-induced disease, can trigger antibody-dependent cell-mediated cytotoxicity (ADCC) (42). Although traditionally, such antibodies have been considered as a useful clearance mechanism for virus infected cells it could also be argued that they may have a damaging role. Hypothetically, shed viral glycoproteins could bind to uninfected cells through specific and even non-specific interaction thus making these cells targets for NK cell-mediated lysis following the binding of specific IgG. The balance between virus-infectivity neutralizing antibodies and ADCC antibodies may therefore be critical in determining the clinical outcome. It has also been suggested that immune complexes formed by non-neutralizing antibodies bound to viral protein may deposit in lung capillaries and cause complement activation and tissue damage, known as antibody-mediated enhanced respiratory disease (ERD) (43, 44). The potential role of the complement system also requires investigating particularly given its linkage to the coagulation pathways.

Serum IgA may also contribute to immunopathology as suggested by its association with disease severity (12, 19, 20). sIgA are able to induce interleukin (IL)-6, IL-8, monocyte chemoattractant protein (MCP)-1 and granulocyte–macrophage colony stimulating factor (GM-CSF) production by normal human lung fibroblasts (45). On the other hand, serum IgA can also mediate anti-inflammatory effects in innate immune cells (46). In serum, IgA exist in two different molecular forms, namely monomeric IgA and polymeric IgA. Both forms exhibit interactions with FcγRI/CD89 to some extent. CD89 is an activating, *γ*-chain associated, Fc receptor for IgA expressed on myeloid cells. CD89 induce phagocytosis of complexed IgA antigens and initiates ADCC (47). Oortwijn et al. (48) demonstrated that the initial interaction of monomeric and polymeric IgA with CD89 is similar. However, monomeric IgA dissociates more rapidly from CD89. In view of the large excess of monomeric IgA in serum, monomeric IgA competes for CD89 interaction with polymeric IgA, thus preventing cell activation initiated by receptor aggregation contributing to anti-inflammatory signals. It may therefore be of interest to investigate, in COVID-19 patients, the association of these two different IgA forms with different clinical outcomes.

## hCoV Cross-Reactive T Cell Memory

There is now also evidence that pre-existing T cell immunity to common cold coronaviruses can prime the response to SARS-CoV-2. Using functional validation of predicted epitopes when arranged in epitope “megapools” and using PBMCs derived from convalescing COVID-19 cases, Grifoni et al. (49) recently reported that all patients studied consistently generated a substantial CD4+/CD8+ T cell response against SARS-CoV-2. In terms of total CD4+ T cell response per donor, on average ~50% of the detected response was directed against the Spike protein, and ~50% was directed against the rest of the SARS-CoV-2 ORFeome. More importantly, when the exact same set of experimental techniques were used with blood samples from healthy control donors (collected between 2015 and 2018) substantial cross-reactive coronavirus T cell memory was detected, suggesting cross-reactive T cell recognition between seasonal cold coronaviruses and SARS-CoV-2. All healthy donors were IgG seropositive to HCoV-OC43 and NL63 RBD, to varying degrees, in line with the endemic nature of these viruses (50–54).

## Lessons from Vaccine Studies in Non-Human Primates

The role of neutralizing antibodies induced by SARS and MERS CoVs S glycoproteins in protection against viral replication in susceptible hosts, including mice, ferrets, hamsters, and macaques (55–57) has been demonstrated in several studies. However, the effect of Spike-specific immunity in protecting against lung injury mediated by immunopathological mechanisms is controversial. Cases of vaccine-induced immunity that help viral clearance and protect mice or ferrets against lethal challenge have been reported (58, 59). On the other hand, in African green monkeys the specific immune memory of SARS-CoV-1 induced by previous infection enhanced lung inflammation following a homologous challenge (60). Using Chinese rhesus macaques, Liu et al. (61) showed the protection of macaques against viral replication by an MVA-based vaccine. However, the same animals showed a concomitant enhancement of acute diffuse alveolar damage suggesting that S-specific immunity promotes Acute Lung Injury (ALI). In addition, in productively infected lungs, anti-Spike IgG caused severe ALI by abrogating a wound-healing macrophage response and TGF-*ß* production while promoting the production of proinflammatory cytokines IL-8 and MCP1 followed by the accumulation of inflammatory macrophages (6). SARS-CoV-1 infection of Chinese macaques is generally characterized by prompt control of viral replication and appearance of mild lung lesions (61). The mechanisms underlying the resistance of Chinese macaques to SARS-CoVs-induced ALI are largely unknown. Liu and colleagues (6) suggest that these effects may be the consequence of a rapid control of viral replication in the lungs, sufficient to create a delay between lung inflammation and high-titer antibody production. Conversely, control of virus replication may be less efficient in SARS patients since the peak in viral load coincides with the first appearance of the antibody response, approximately 10 days after the onset of symptoms (16). Consistent with these conclusions, previous administration of anti-S-IgG in Chinese macaques leads to acute lung injury due to a massive accumulation of monocytes/macrophages in the lungs (6).

## Discussion

Based on the available data on COVID-19 patients and data from the previous SARS-CoV-1 and MERS outbreaks, there is substantial evidence that cross-reactive B and T cell responses may establish an unfavorable environment for the primary immune response to SARS-CoV-2 virus. We have summarized here increasingly available evidence that supports this hypothesis, including several reports of unconventional and accelerated antibody responses associated with disease severity in COVID-19, SARS, and MERS patients (15, 22–24) as well as evidence of hCoV cross-reactive antibody responses in SARS and COVID-19 (24–26, 33, 34). Additional evidence (32) highlights that human antibody responses to SARS-CoV-2 and other spillover CoV share similar modalities and are both characterized by a unique skewing of the B cell repertoire with an early recruitment of B cells expressing a limited subset of V genes without significant somatic mutations.

There is also evidence that pre-existing T cell immunity to common cold coronaviruses can prime the response to SARS-CoV-2 and that this priming is skewed to spike (49).

**Figure 1:**
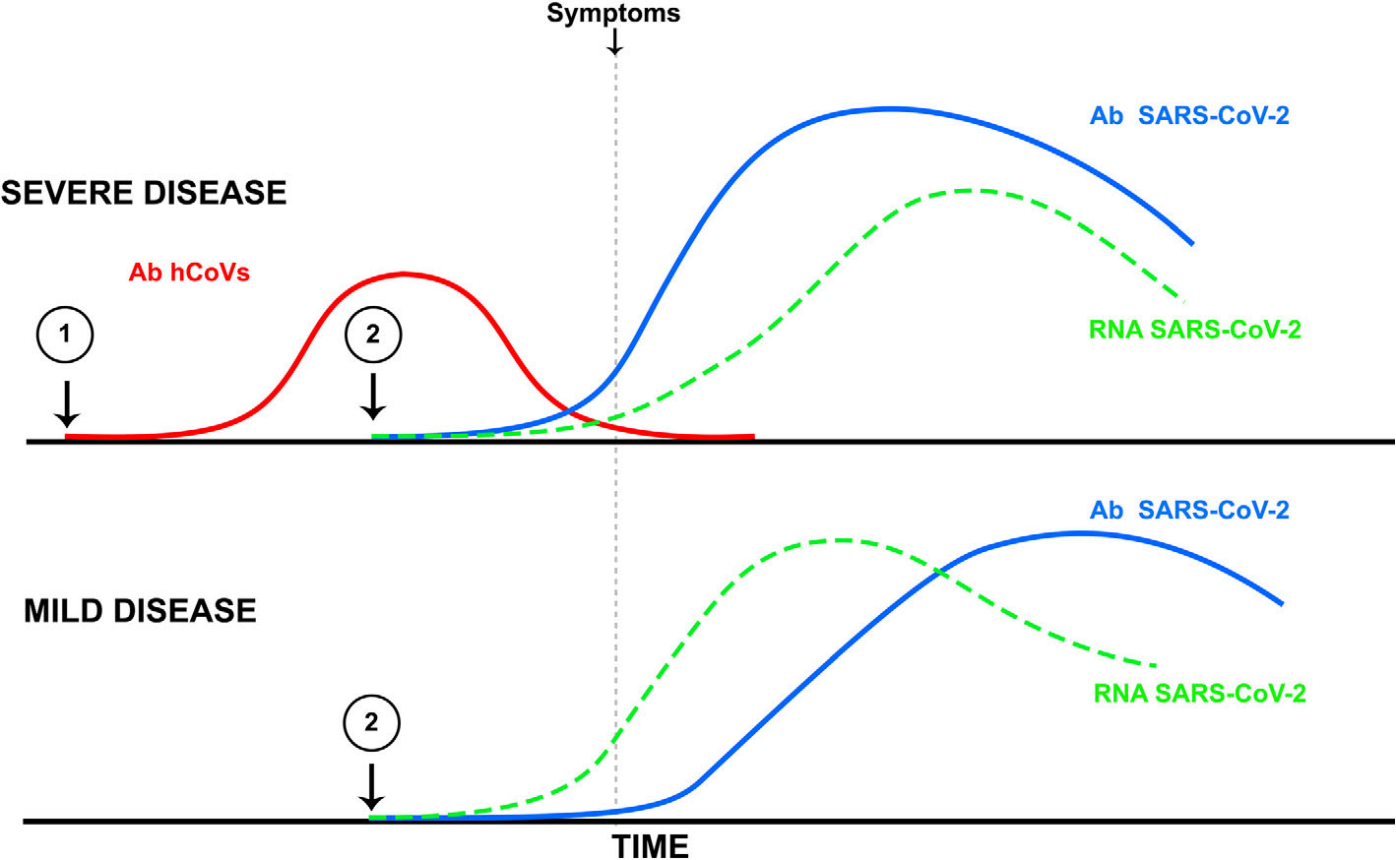
Illustrates the potential effects of an antibody response to infection by a common human coronavirus (Ab hCoVs, red line point 1) generated prior to SARS-CoV-2 infection and the induction of SARS-CoV-2 antibodies (blue line point 2) that cross-react with hCoVs. According to this hypothesis, in the absence of a previous hCoV infection, the antibody response to SARS-CoV-2 would follow a slower kinetics with a peak following that of viral replication, thus inducing a mild to moderate disease (lower panel). In contrast, a pre-existing antibody response to hCoVs would be followed by a very rapid (anamnestic) increase in SARS-CoV-2 antibodies that would precede the peak of viral replication (upper panel), thus inducing conditions for antibody-dependent enhancement or immunopathology by other antibody-dependent mechanisms leading to severe disease.

It remains to be determined whether immune cross-reactivity can trigger immunopathogenesis in COVID-19 patients (**Figure 1**). However, there is sufficient evidence for an antibody enhancement role in both SARS-CoV and MERS-CoV infections. Furthermore, in studies on atypical measles and dengue hemorrhagic fever, as well as in several respiratory diseases, including RSV and pandemic influenza, the worsening of the disease following vaccination has been reported (62, 63).

The observation of Liu et al. (6) on the association of an early antibody response occurring prior to viral clearance with abrogated wound-healing responses and increased inflammatory macrophage-infiltration into the lungs of Chinese rhesus macaques may be instrumental in understanding the potential role of an accelerated cross-reactive immunity in the pathogenesis of COVID-19. Within this framework, cross-reactivity priming with common hCoVs, sustained by long-lasting T cell immunity (49) may be responsible for the unconventional antibody responses observed in COVID-19 patients, with IgG appearing sooner than IgM in severe cases. Antibody enhancement may play a role in this context, exacerbating the inflammatory response generated by virus internalization in macrophages mediated by Fc*γ* Receptors (Fc*γ*R) and/or by other mechanisms.

Tetro (64) suggested that a biological mechanism underlying the geographical differences in COVID-19 pathogenesis may be linked to individuals primed by one or more previous exposures to coronaviruses, and due to the heterogeneity of the antigenic epitopes, may experience the effects of ADE.

Disease augmentation by previous exposure to seasonal cold coronaviruses may also partially explain the drop in the case fatality rate (CFR) observed recently in the Lombardy region in Italy since the end of March (https://www.epicentro.iss.it/coronavirus/).

The low incidence of COVID-19 in children seems to contradict the hypothesis of immunopathogenesis requiring priming with hCoVs, since hCoV infections are common in children (65). However, the different susceptibility of children to SARS-CoV-2 morbidity may be due to differences in composition and functional responsiveness of their immune system (66). The presence of natural antibodies (67), which are produced by innate memory B cells, a cell population that is generated independently of the germinal centers and is most abundant in children, may contain the infection during the 2 weeks necessary for the production of high-affinity antibodies. The milder disease in children might also be related to “trained immunity” (68) which represents an innate immune “memory” response (69). Another contributing difference may be the lower expression of the ACE2 receptor of SARS-CoV-2 in children (70). On the other hand, the finding that SARS-CoV-2 infected children with a multisystem inflammatory syndrome (MIS-C) have a dysregulated humoral immune response characterized by serological reactivity to hCoVs and other respiratory viruses, provide additional evidence of the immunopathological role of cross-reactive immunity.

The potential role of early antibody responses in the triggering of immunopathogenesis has been discounted by several authors based on the observation that plasma derived from convalescent patients (both in SARS and COVID-19) has beneficial effects (71–73). However, the possibility that the early antibody response found associated with severe cases versus the late response in convalescent patients may be sustained by different types of antibodies should be considered. With the same reasoning, the selection of convalescent donor plasma based on the titration of neutralizing antibodies and the timing of treatment should be carefully considered in the ongoing clinical trials (21).

Finally, the immunopathogenesis potential of cross-reactive immunity should be considered in current efforts to develop safe and effective vaccines. Careful selection of antigenic B cell epitopes will be necessary to avoid the potential induction of antibody dependent enhancement of disease. In addition, vaccination strategies that induce airway memory CD4+ T cells targeting conserved epitopes could be safer and have broader applicability in the context of COVID-19 and other respiratory virus epidemics (74).

## Data Availability Statement

The original contributions presented in the study are included in the article; further inquiries can be directed to the corresponding author.

## Author Contributions

All authors listed have made substantial, direct, and intellectual contribution to the work and approved it for publication.

## Funding

This work was supported by FUR 2019, Department of Neuroscience, Biomedicine and Movement Sciences, University of Verona, by Department of Excellence 2018/2022, MIUR, Italy, and by the Brain Research Foundation Verona (BRFV).

## Conflict of Interest

Author AB was employed by the company Solongevity Research.

The remaining authors declare that the research was conducted in the absence of any commercial or financial relationships that could be construed as a potential conflict of interest.
